# Effects of lubricant-fuel mixing on particle emissions in a single cylinder direct injection spark ignition engine

**DOI:** 10.1038/s41598-021-03873-w

**Published:** 2022-01-07

**Authors:** Hoseung Yi, Jihwan Seo, Young Soo Yu, Yunsung Lim, Sanguk Lee, Jongtae Lee, Hanho Song, Sungwook Park

**Affiliations:** 1 Department of Mechanical Convergence Engineering, Hanyang University, 222 Wangsimri-ro, Seongdong-gu, Seoul, 04763 Republic of Korea; 2 National Institute of Environmental Research, 42 Hwangyong-ro, Seo-gu, Incheon, 22689 Republic of Korea; 3 Department of Mechanical Engineering, Seoul National University, 1 Gwanak-ro, Gwanak-gu, Seoul, 08826 Republic of Korea; 4 School of Mechanical Engineering, Hanyang University, 222 Wangsimri-ro, Seongdong-gu, Seoul, 04763 Republic of Korea

**Keywords:** Engineering, Mechanical engineering

## Abstract

Gasoline direct injection (GDI) engines emit less carbon dioxide (CO_2_) than port fuel injection (PFI) engines when fossil fuel conditions are the same. However, GDI engines emit more ultrafine particulate matter, which can have negative health effects, leading to particulate emission regulations. To satisfy these regulations, various studies have been done to reduce particulate matter, and several studies focused on lubricants. This study focuses on the influence of lubricant on the formation of particulate matter and its effect on particulate emissions in GDI engines. An instrumented, combustion and optical singe-cylinder GDI engine fueled by four different lubricant-gasoline blends was used with various injection conditions. Combustion experiments were used to determine combustion characteristics, and gaseous emissions indicated that the lubricant did not influence mixture homogeneity but had an impact on unburned fuels. Optical experiments showed that the lubricant did not influence spray but did influence wall film formation during the injection period, which is a major factor affecting particulate matter generation. Particulate emissions indicated that lubricant included in the wall film significantly affected PN emissions depending on injection conditions. Additionally, the wall film influenced by the lubricant affected the overall particle size and its distribution.

## Introduction

Gasoline direct injection (GDI) engines are spark ignited internal combustion engines that inject highly pressurized fuel directly into the combustion chamber. This injection system gives GDI engines several advantages compared to port fuel injection (PFI) engines, which inject fuel into the intake manifold of the engine, when the same fossil fuel is used. One advantage is that the direct injection system increased fuel atomization and vaporization rate, causing a charge cooling effect in the combustion chamber during injection. This allowed GDI engines to reach a higher compression ratio and increase volumetric efficiency relative to PFI engines. Another advantage was that the direct injection system gave the ability to precisely control the amount of fuel at a certain engine operating condition. This led to an increase in overall fuel efficiency compared to another configuration. Due to these advantages, GDI engines have better fuel economy and higher power output, producing fewer carbon dioxide (CO_2_) emissions than PFI engines^1^. Despite these advantages, GDI engine particulate emissions were significantly higher than PFI engines, as has been shown in many studies^2–6^. In addition, several studies reported that GDI engines emitted more PN^7,8^ and ultrafine particles^2,9^ than diesel engines equipped with a particulate filter. Since it is well known that ultrafine particles have a detrimental impact on the atmosphere and human respiratory systems^10,11^, stringent regulations to reduce particulate emissions on vehicles have been established. Starting in 2009, regulation of particulate mass (PM) for GDI engines was legislated, and regulations on PN were enacted in 2014 in Europe. Furthermore, EURO-6c has been implemented in 2017, which regulates PM and PN levels in GDI engines by requiring them to be at the same levels as diesel vehicles. In the U.S., the California Low Emission Vehicle II (LEV II) program required gasoline vehicles to comply with the PM standard starting in 2008. Later, the LEV III program was legislated to further restrict PM emissions regardless of fuel.

To meet the regulations, various studies have been conducted to find the cause of PN to reduce particulate emissions. Among them, several studies concentrated on lubricant consumption in the combustion chamber. Lubricant in the engine has various functions including: reducing friction between surfaces, cleaning soot and sludge in the engine, preventing rust and corrosion, cooling parts such as the piston, and sealing gaps to prevent combustion gas from escaping. However, these characteristics also caused entrainment of lubricant into the combustion chamber through several paths such as the piston ring, valve seals, blow-by gas, and turbochargers^12,13^, which resulted in the consumption of lubricant. Even though the consumption of lubricant was marginal, accounting for only about 0.1% in modern engines, it contributed significantly to particulate emissions in some cases^14^. Sonntag et al.^15^ estimated that the contribution of lubricant to PM emission rates is 25% in light-duty gasoline vehicles. In addition, metal additives in the lubricant (which are included to enhance the performance of the lubricant) greatly affected particulate emissions^16^. Due to these issues, several studies have been performed to identify the effect of lubricant consumption on particulate emissions. For example, Miller et al.^17^ modified a diesel engine fueled with hydrogen to remove fuel-derived soot. The results showed that most particles consisted of metals with some organic compounds and a marginal amount of elemental carbon. Christianson et al.^18^ compared two light duty vehicles with two different ethanol blend ratio fuels. Results indicated that both the number and size of particles decreased during the break-in period of the lubricant, but they are expected to increase due to lubricant aging. Pirjola et al.^19^ examined particulate emissions on five lubricants with different properties using a turbocharged GDI vehicle. From the results, PN was significantly affected by the properties of the lubricant and metallic additives included in the lubricant. Amirante et al.^20^ investigated the contribution of lubricant to particulate emissions by implementing different injection methods using a spark ignition engine equipped with both PFI and GDI injector fueled by gasoline or natural gas. The results showed that the addition of lubricant significantly increased particulate emissions in the lowest size range regardless of the methods and size range over 50 nm was observed when the lubricant directly entered the combustion chamber.

As shown above, these studies focused on the effect of lubricant on particulate emissions but did not analyze how the engine lubricant influenced particulate matter in a GDI engine. Before illustrating the relationship between particulate matter and lubricant, detailed information related to particulate formation is required. Particulate matter formed in a GDI engine is mainly due to inhomogeneous air–fuel mixture and the existence of wall film^21^. Inhomogeneous mixtures appear when the fuel does not properly mix with air in the combustion chamber, resulting in partial fuel-rich zones. Then, incomplete combustion occurs in fuel-rich zones due to a lack of air during combustion, which leads to the formation of particulate matter^22^. A wall film develops when fuel spray impinges on the piston or in-cylinder liner, creating thin fuel film. This fuel film does not evaporate and exists during combustion, and it can cause diffusion flame from incomplete combustion, leading to particulate matter formation^23^. This process is similar to lubricant entrainment and lubricant emission due to mixture formation in a GDI engine, which have been investigated by Gohl et al.^24^. Hence, studying the lubricant behavior can provide detailed insight about how lubricant influences particulate matter formation during the combustion phase. Therefore, we investigated the influence of lubricant on the formation factor of particulate matter and its effect on particulate emissions in a GDI engine by utilizing combustion and an optical engine with various injection conditions fueled with different ratios of lubricant blended gasoline.

## Experimental methods

To analyze the influence of lubricant on particulate matter formation and its effect on particulate emissions in direct injection spark ignition engine with respect to lubricant blend ratio, engine experiments were conducted with various mix ratios.

### Combustion engine experiment apparatus

The engine used in this experiment is a four stroke, single cylinder GDI engine. Detailed specifications of this engine are shown in Table 1. The fuel injection system of this engine is a side mounted, wall guided six-hole injector located between two intake valves. The ignition system of this engine is a spark plug mounted and positioned on center of the cylinder head.

**Table 1:** Engine specifications.

Item	Specification
Bore	77 mm
Stroke	85.44 mm
Displacement volume	397.86 mL
Compression ratio	10.5:1
Intake valve open/close	BTDC 345/95°
Exhaust valve open/close	ATDC 108/346°

**Figure 1:**
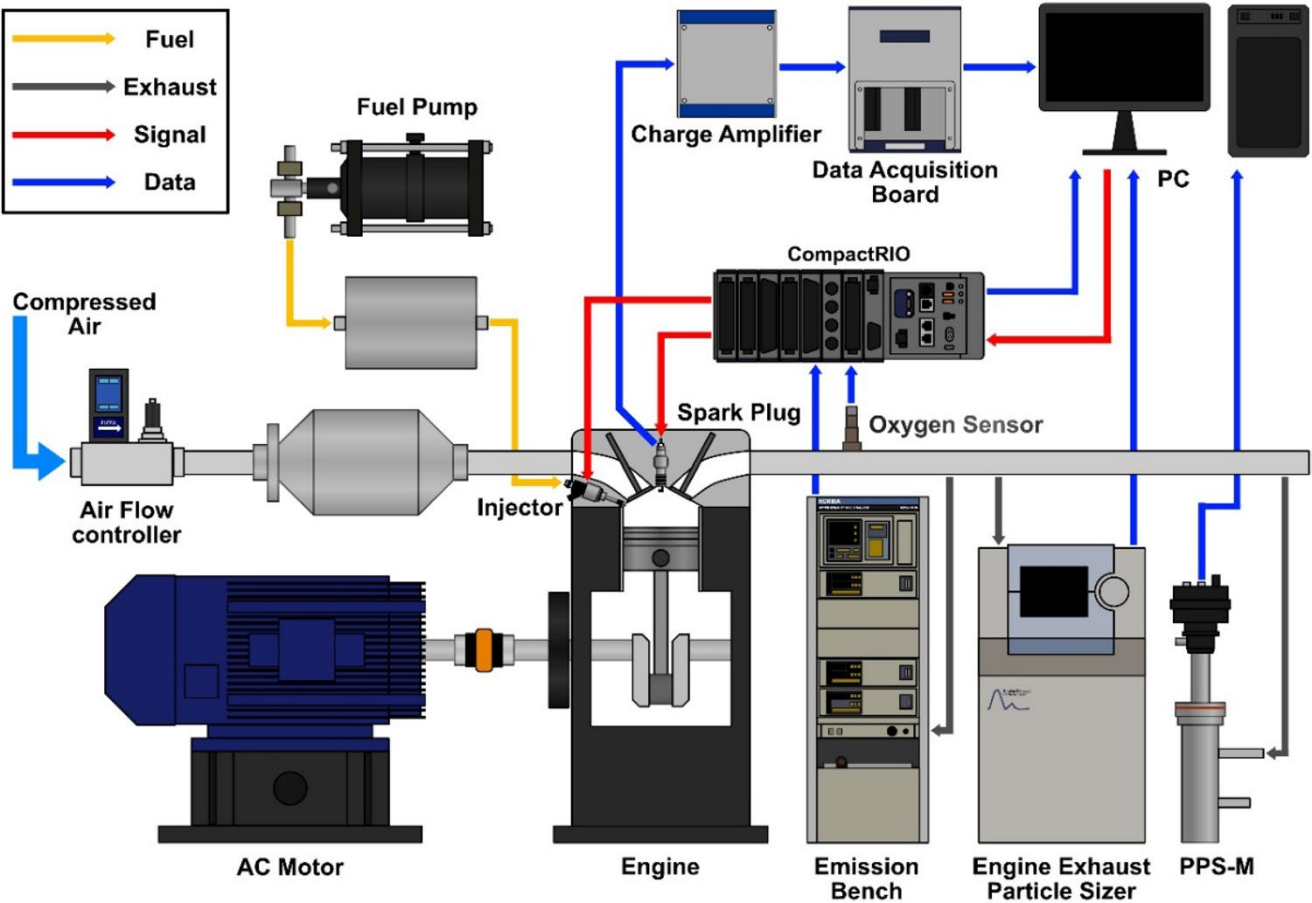
Schematic diagram of engine operating systems and exhaust emission measuring systems.

For all test conditions, the engine was operated using an alternating current (AC) motor, which kept the engine speed constant during the experiment. The intake air was constantly provided into the intake port using compressed air and a mass air flow controller. Fuel was pressurized using an air-driven liquid pump, and pressure fluctuation was stabilized by including a fuel chamber between the pump and injector. Injection timing and spark timing were controlled using a CompactRIO (National Instruments) system and the LabVIEW (National Instruments) program. Equivalence ratio was measured using a broadband lambda sensor (LSU4.9, Bosch), and it was used as a feedback signal to control injection duration. In-cylinder pressure was acquired using a piezoelectric pressure sensor integrated into a spark plug (6115B, Kistler), and the signal was amplified by the charge amplifier (Kistler). This signal was then sent to the data acquisition board (USB X series, National Instruments) for post-processing. In-cylinder pressure data were measured over 300 cycles and averaged to minimize cyclic variation. Measurement of total hydrocarbon (THC), carbon monoxide (CO), and nitrous oxides (NOx) emitted from the engine exhaust were performed using a MEXA 9100 (Horiba) emission bench. Engine exhaust particle number (PN) and size distribution were measured using PPS-M (Pegasor) and EEPS 3090 (TSI), respectively. After a sufficient duration of stabilization, emission data were collected in real time for over three minutes when each test condition was at steady state. Data regarding combustion characteristics and engine power (kW) were post processed using data acquired from cylinder pressure, crank angle, and intake/exhaust pressure. Emission data were post processed to be in gram per kilowatt-hour (g/kW-hr) for gaseous emissions and numbers per kilowatt-hour (#/kW-hr) for particle number by utilizing engine power. Intake flow rates were taken from experiment data, and the density of gaseous substances in exhaust emissions was based on the book ‘An Introduction to Combustion’^25^. Particle size distribution was post processed into concentration (%) by dividing each particle size number by the sum of all particle size numbers. The overall experimental setup mentioned above is illustrated in Fig. 1.

### Optical engine experiment apparatus

**Figure 2:**
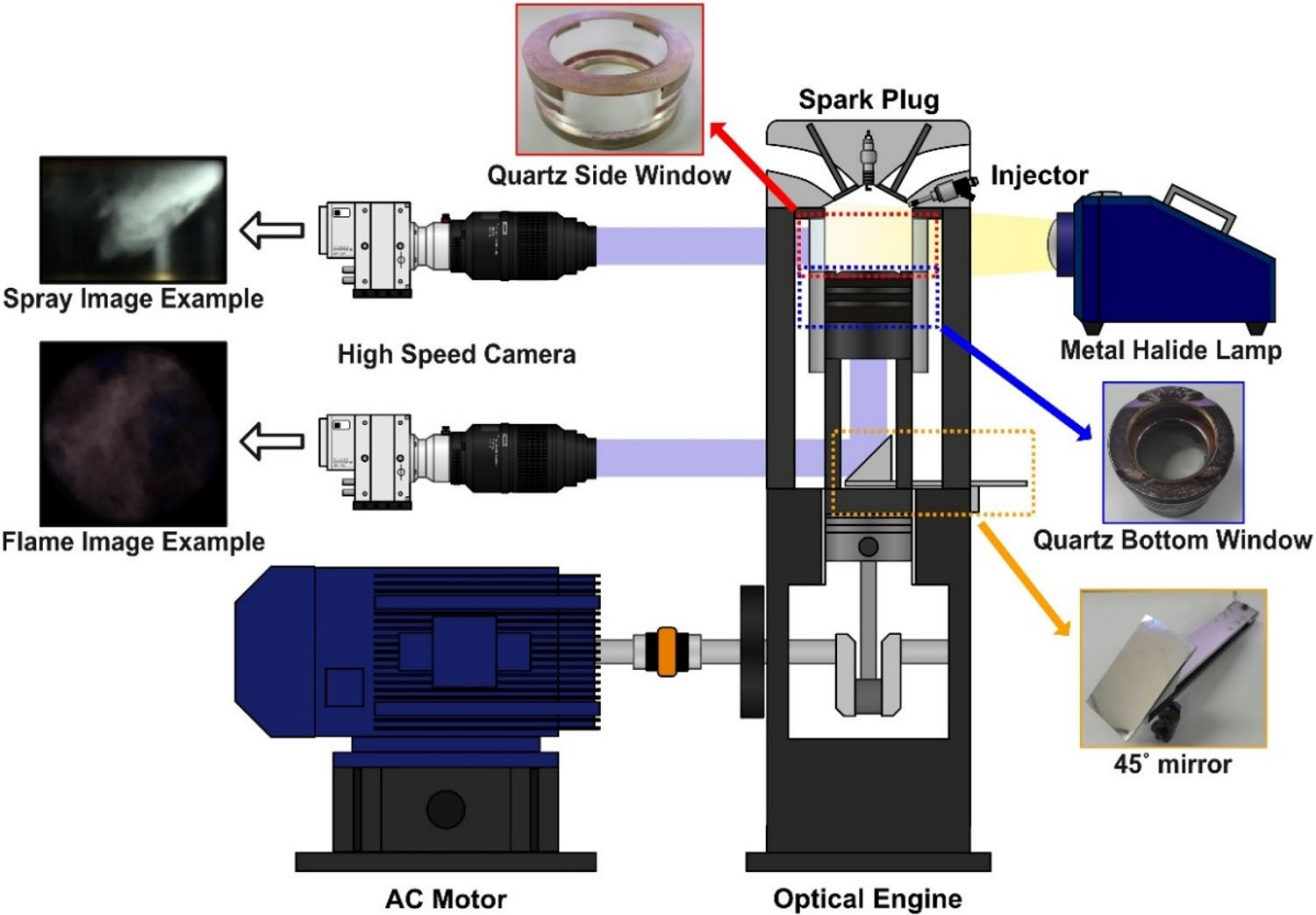
Schematic diagram of optical engine for spray and flame visualization.

In-cylinder fuel spray and flame visualization were conducted by implementing an optical engine system in between the cylinder head and body as shown in Fig. 2. Images of the fuel spray were captured on the side of the engine by adding a quartz side window between the cylinder head and liner, which has an optical accessible width of 49 mm and a height of 34 mm. A metal halide lamp was used as a light source to observe the development of spray by the Mie-scattering method. Thirty cycles of spray image were continuously recorded since the interactions between spray behavior, in-cylinder flow and turbulence are not same for all cycles. All spray images were recorded with the same image resolution, exposure rate, focal point, and aperture. Also, camera was calibrated using the provided camera control program (PCC 3.1, Phantom) to acquire same image intensity for all cases. Then, the recorded spray image was cropped to only see the spray visualized by the side quartz window, and it was post processed to average the spray image to reduce cycle-to-cycle variation. Additionally, the average spray image was converted into a color map to clearly visualize spray brightness. The flame image was captured on the bottom of the engine through a 45° mirror and a quartz bottom window located at the piston head with an optical accessible diameter of 55 mm. No light source was used for the flame image to capture the chemiluminescence emitted from the flame. Over 120 cycles of flame images were taken because the diffusion flame is affected by spray behavior, in-cylinder flow, film formation, and flame propagation. All flame images were recorded with the same image resolution, exposure rate, focal point, and aperture. Also, camera was calibrated using the provided camera control program (PCC 3.1, Phantom) to acquire same image intensity for all cases. Recorded flame images were cropped to view the diffusion flame visualized by the bottom quartz window and to remove reflected lights formed on the bottom of the quartz window. All visualization images were obtained using a high-speed camera (VEO 710, Phantom) at 9000 frames per second.

### Combustion engine experiment conditions

The combustion engine was operated at 1500 rpm, and the coolant temperature was set to 80 ℃. An intake flow rate of 130LPM was set to ensure the engine had medium load, which prevents knocking caused by auto-ignition of the lubricant^26^. Injection duration was set to maintain the equivalence ratio to stoichiometric for all test conditions. Spark timing was set by the maximum brake torque (MBT) of each test condition. Injection pressure varied from 10 MPa up to 35 MPa, and injection timing varied from BTDC 330° to BTDC 180° to create the particulate matter formation conditions and check the influence of the lubricant. Test conditions mentioned above are organized in Table 2.

**Table 2:** Engine operating conditions.

Item	Specification
Engine speed	1500 rpm
Intake air flow rate	130LPM
Coolant temperature	80 °C
Equivalence ratio	1.0
Injection pressure	10, 20, 35 MPa
Injection timing	BTDC 330, 270, 180°
**Spark timing**	
Combustion	MBT
Optical	Set from combustion results
**Injection duration**	
Combustion	Set by equivalence ratio
Optical	
Spray image	1.0, 1.5, 2.0 ms
Flame image	Set from combustion results

### Optical engine experiment conditions

For optical engine conditions, engine rotation speed, coolant temperature, intake flow rate, injection pressure, and injection timing were set to be the same as the combustion engine experiment conditions. Injection duration for spray visualization was set from 1.0 to 2.0 ms for all test conditions. Injection duration and spark timing for flame visualization were set according to the results from the combustion experiment. The test conditions mentioned above are also organized in Table 2.

### Fuel composition conditions for combustion and optical engine experiments

For both the combustion and optical engines, SAE 5W20 lubricant (Hyundai Mobis) was blended into commercial gasoline produced for summertime use (GS Caltex) in different weight percentages varying from zero to five weight percent (wt%). Gasoline, lubricant, and gasoline-lubricant blended fuel property measurement tests were requested from the Korea Petroleum Quality and Distribution Authority. Detailed percentage ratios of the blended fuel and properties of lubricant and gasoline are listed in Table 3. Here, “G100” denotes 100wt% of gasoline is used and “GxxLy” denotes “xx” wt% of gasoline and “y” wt% of lubricant is blended. For example, “G97L3” denotes 97wt% of gasoline and 3wt% of lubricant was blended. A maximum of G95L5 was used since the exhaust measurement systems used in this experiment could not measure the emission data of blended fuel that had more than 5wt% lubricant.

**Table 3:** Fuel blend ratio and properties of fuel components.

		Gasoline (G100)	Lubricant (O100)	G95L5
Properties	Lower heating value [J/g]	42,740	42,910	-
	Kinematic viscosity [mm^2^/s @20 °C]	0.5330	128.5	0.6403
	Absolute viscosity [mPa*s @25 °C]	0.3673	77.34	0.4235
	Density [kg/m^3^ @15 °C]	718	851.1	718.8
	Initial boiling point [°C]	36.6	326.1	–
	Final boiling point [°C]	193.7	610.7	–
	Vapor pressure [kPa @ 37.8 °C]	57.4	(Unable to measure)	53.8

## Results

### Combustion engine experiments on gasoline-lubricant blend ratio

**Figure 3:**
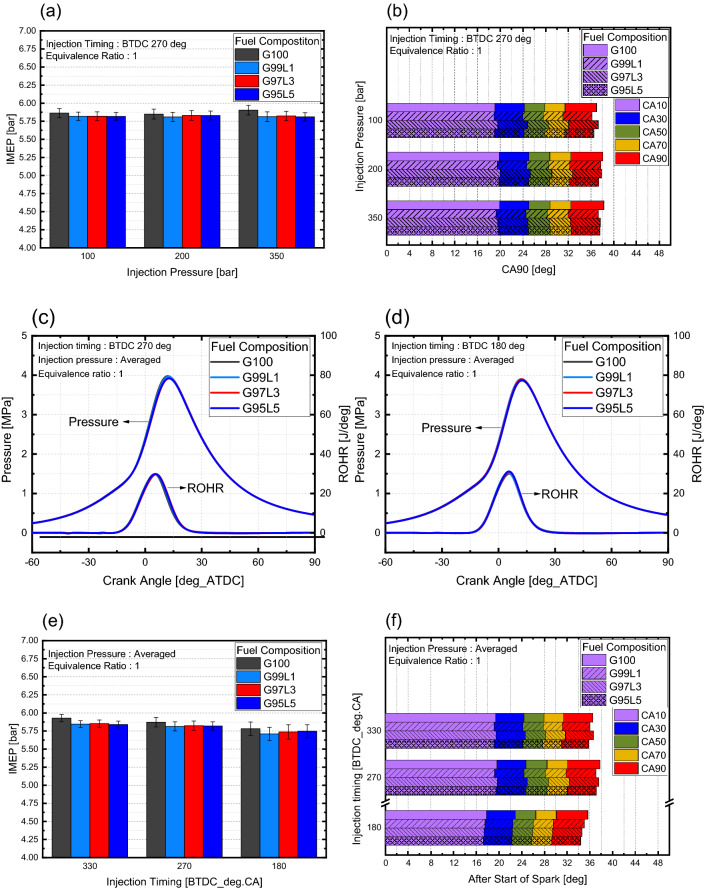
Combustion characteristics with respect to lubricant blend ratio.

**Figure 4:**
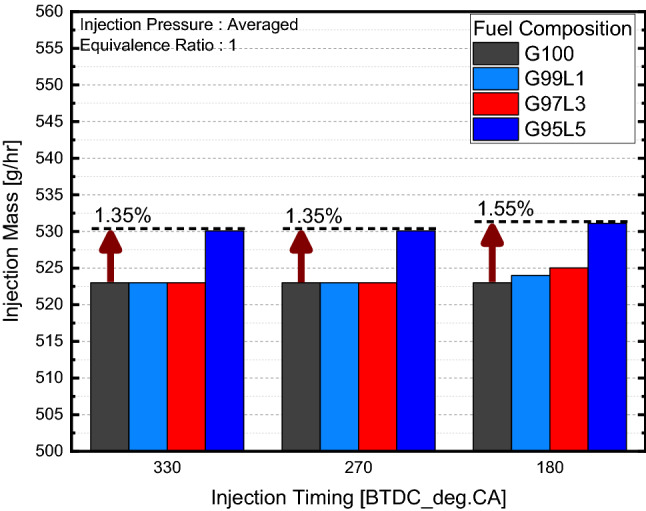
Fuel consumption with respect to lubricant blend ratio.

Changes in combustion characteristics at different injection pressure with respect to blend ratio were observed as shown in Fig. 3a,b. Since the combustion results were similar on all three injection pressures, data with respect to injection pressure were averaged to observe overall results. When observing the results of in-cylinder pressure shown in Fig. 3c, the lubricant blend ratio had a negligible effect on in-cylinder pressure and ROHR regardless of injection timing. Including lubrication oil seemed to slightly reduce IMEP compared to G100 for all injection timings, but the difference was within the error margin and there were no significant differences between different blend ratios over 1 wt% as shown in Fig. 3e. Here, combustion duration, calculated from ROHR, is defined as crank angle duration from 10 to 90% of mass fuel to burn. This means that combustion duration can be interpreted as combustion speed. The combustion duration displayed in Fig. 3f showed no significant differences in lubricant blend ratio for injection timings of BTDC 330 and 270°. For an injection timing of BTDC 180°, combustion duration seemed to decrease when the lubrication blend ratio increased. However, when inspecting the ROHR at an injection timing of BTDC 180° presented in Fig. 3d, the results were similar for all blend ratios, which indicated that the combustion duration differences were marginal. From these results, the combustion characteristics indicated that lubricant blend ratio did not affect mixture homogeneity since mixture homogeneity impacts flame propagation. Fuel consumption with respect to lubricant blend ratio presented in Fig. 4 was calculated based on the injection duration and injection quantity specification of the injector. As the lubricant blend ratio increased, the fuel consumption also increased to an average of 1.4% as compared to G100. This result implies that excess fuel did not participate in the combustion process but remained unburned. Because the equivalence ratio was a fixed experimental condition, combustion characteristics were similar for all cases, and the lower heating values (LHV) were similar for the gasoline and lubricant.

**Figure 5:**
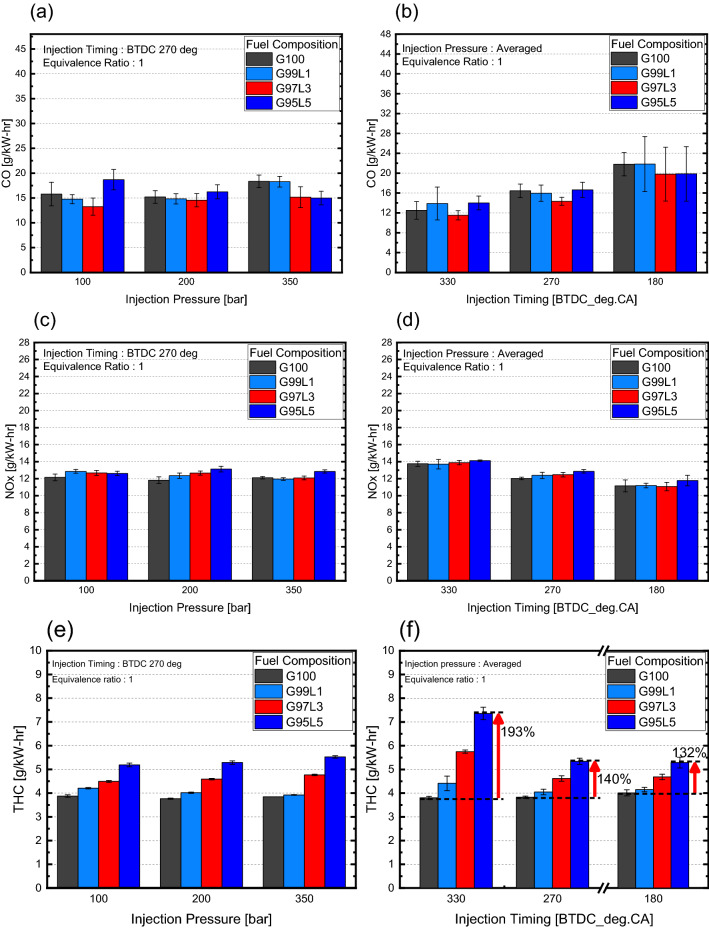
Gaseous emissions with respect to lubricant blend ratio.

CO emissions shown in Fig. 5a had no trend with respect to lubricant blend ratio for all injection pressures therefore data was averaged to see overall trend at different injection timing. Even when the data was averaged, CO emissions had no trend on all injection timings as shown in Fig. 5b. NOx emissions shown in Fig. 5c had similar trend for all injection pressures therefore was averaged to observe overall trend at different injection timing. Averaged NOx emissions showed small differences with lubricant blend ratio as illustrated in Fig. 5d, and the trend was similar for each injection timing. Here, NOx emissions are mainly influenced by in-cylinder pressure since the combustion temperature, the main factor for NOx emissions, is proportional to in-cylinder pressure and combustion speed^27^. From this knowledge, the slight difference in NOx emissions was due to measurement error since there were no differences in in-cylinder pressure, and the trend in NOx emissions showed no relationship between combustion speed and lubricant blend ratio. Generally, CO emissions are produced due to incomplete combustion from a rich air–fuel mixture or an inhomogeneous mixture. NOx emissions are produced by the factors mentioned above and from lean air–fuel mixtures^27^. Since CO and NOx emissions were not dependent on lubricant blend ratio for all injection timings and combustion was stoichiometric for this experiment, we concluded that the lubricant blend ratio did not have an impact on mixture homogeneity. On the other hand, the gasoline-lubricant blend ratio had a significant effect on THC emissions displayed in Fig. 5f. Since THC emissions trend with respect to lubricant blend ratio at different injection pressures were similar as shown in Fig. 5e, data was averaged to observe overall trends regarding on injection timing. For an injection timing of BTDC 330°, THC emissions increased significantly as the lubricant blend ratio increased, resulting a 193% increase when comparing the emissions of G100 and G95L5. This increase was notably higher than other injection timings. When injection timing was delayed to BTDC 270°, the difference between G100 and lubricant blended fuel was reduced, leading to a 140% increase in emissions between G100 and G95L5. As injection timing was retarded further to an injection timing of BTDC 180°, the maximum difference between G100 and lubricant blended fuel was slightly reduced to a 132% increase in emissions between G100 and G95L5. THC emissions are generated by several factors, including a rich air–fuel mixture, incomplete combustion, and unburned fuels^27^. From the experimental conditions and previous results, rich air–fuel mixtures and incomplete combustion were not factors in this experiment. Thus, we concluded that the increase in THC emissions were mostly due to an increase in unburned fuels.

### Optical engine experiments on gasoline-lubricant blend ratio

**Figure 6:**
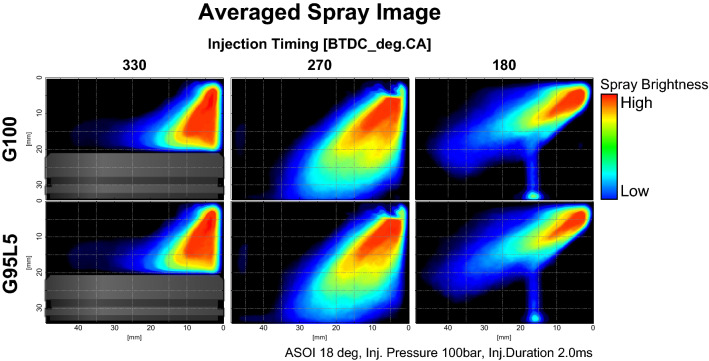
Averaged spray image with respect to lubricant blend ratio.

**Figure 7:**
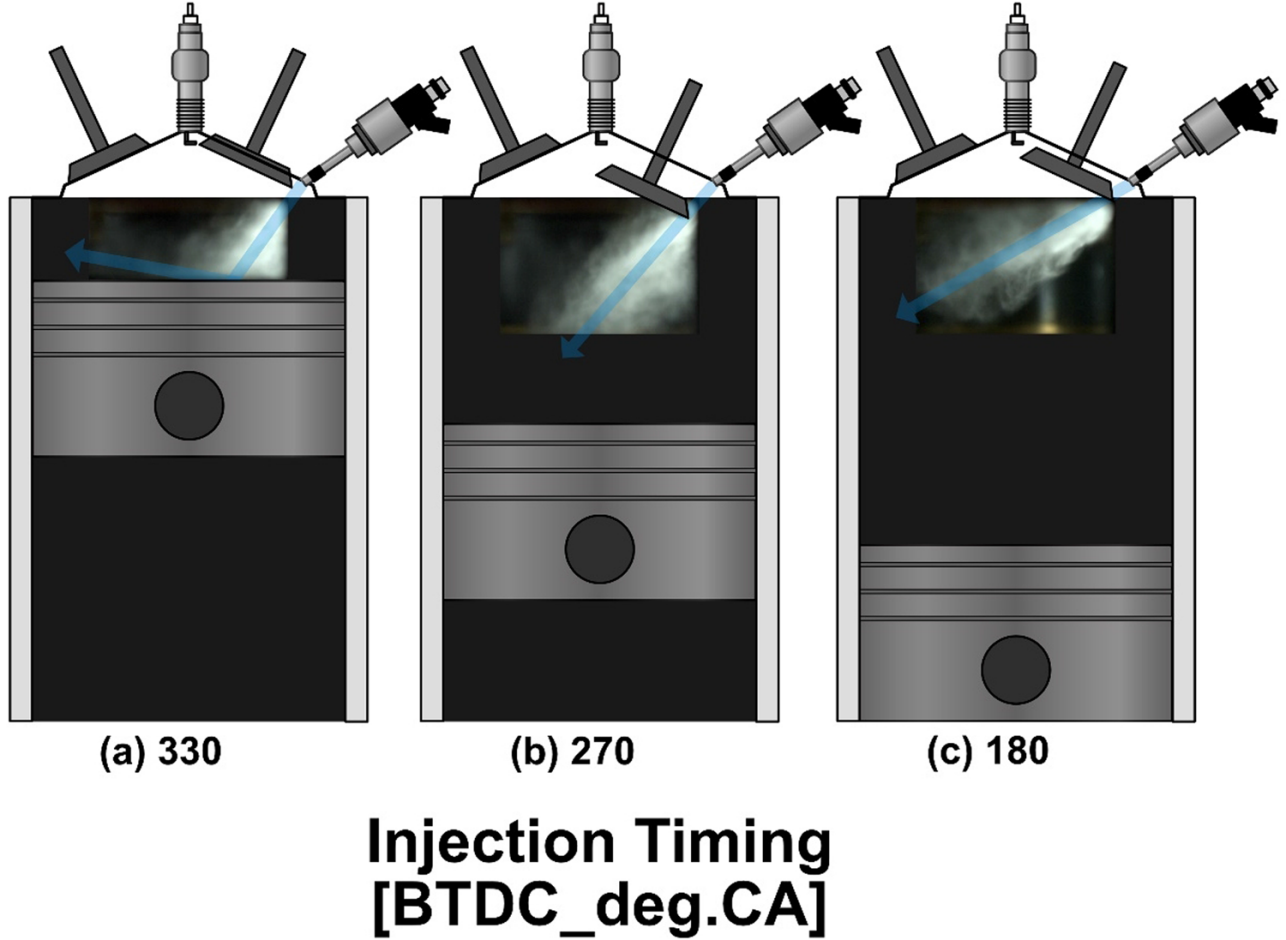
Schematic descriptions of piston and intake valve positions with respect to injection timing and spray penetration direction.

To investigate the effect of lubricant blend ratio, differences in the spray behavior and penetration length at various lubricant blend ratios were measured under an injection pressure of 10 MPa at ASOS 18° (where the injection process has just terminated) by comparing spray images of G100 and G95L5 as presented in Fig. 6. All images were set in a grid to clearly compare the differences in spray images. Furthermore, for an injection timing of BTDC 330°, an illustration of the piston was inserted to demonstrate the interaction between the piston surface and spray. At an injection timing of BTDC 330° (where spray collides with the piston surface and in-cylinder flow increases as illustrated in Fig. 7a), there were no differences in the average spray behaviors and penetration length of the two fuels. Further, the spray behaviors after colliding with the piston were similar. Figure 7b shows the spray image on an injection timing of BTDC 270° where the in-cylinder flow was strong resulting in a downward direction of spray penetration. As shown, slight differences in behaviors were observed, but these were mainly due to the variance in in-cylinder flow. Overall spray penetration lengths were similar. The injection timing of BTDC 180° (where in-cylinder flow was weak leading to an upward spray direction as illustrated in Fig. 7c) also showed similar spray behavior and penetration length between G100 and G95L5. These observations can be supported by the properties of G100 and G95L5 mentioned in Table 3. Density and vapor pressure are fuel properties that influence spray behavior. As density increases, the momentum of the spray increases, resulting in a longer spray penetration length. Vapor pressure has an effect on spray behavior since a higher vapor pressure enhances fuel evaporation, resulting in a reduction in fuel droplet size. As a result, the spray image brightness decreases. However, the difference in density and vapor pressure between G100 and G95L5 were insignificant, meaning that the lubricant blend ratio did not affect spray behaviors and penetration. This result also implies that lubricant blend ratio did not have an impact on mixture homogeneity since spray behavior and penetration greatly impact air–fuel mixture formation^28^.

**Figure 8:**
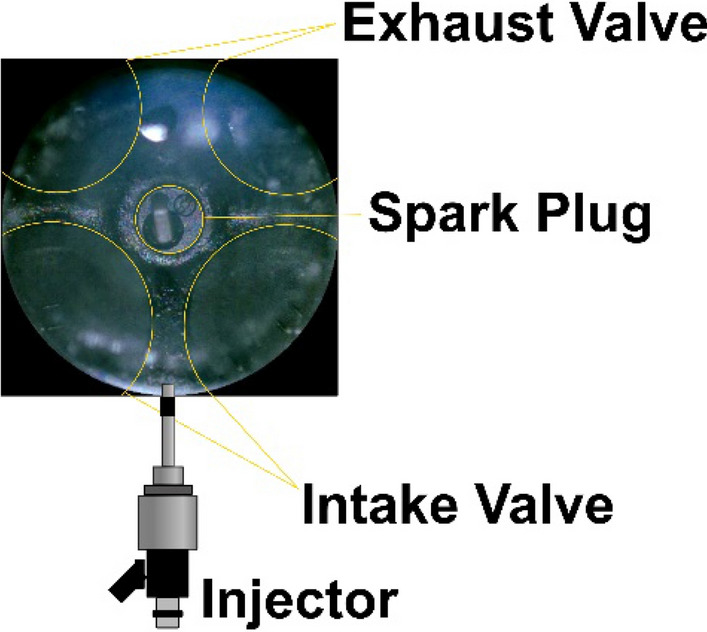
Schematic of valve, spark plug, and injector position for flame image.

**Figure 9:**
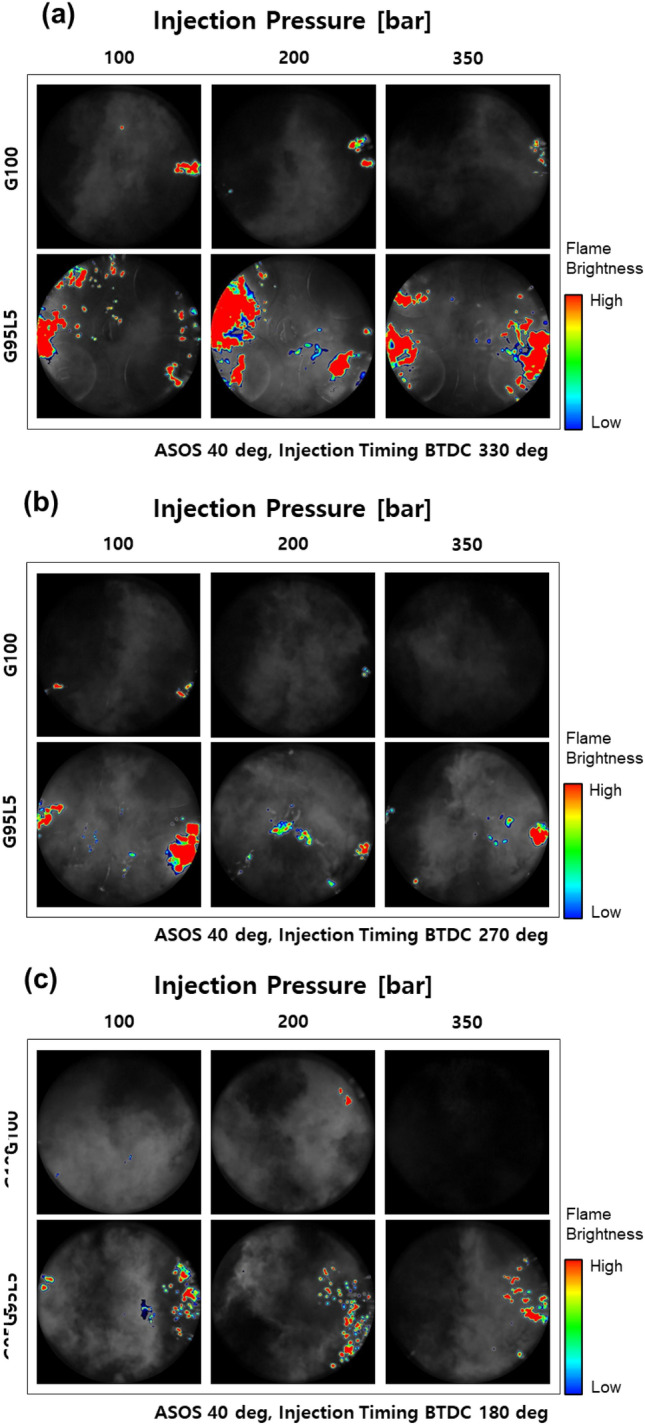
Diffusion flame image with respect to lubricant blend ratio.

To inspect the amount and position of the diffusion flame generated during combustion, the flame images were observed for various lubricant blend ratios under various injection pressures and injection timings. Flame images were also observed at ASOS 40°, where the flame front meets the piston surface or the cylinder liner. One image was chosen from over 120 cycles of images for all cases. Selection of the image was conducted by the following procedures: flame images exhibiting diffusion flames were chosen and post processed to calculate the total area and position of diffusion flame of each image. Calculated data were averaged, and the image closest to the average was chosen and post processed to clearly visualize the area and position of diffusion flame. The position of the intake and exhaust valve, spark plug, and injector can be identified from Fig. 8. At an injection timing of BTDC 330° shown in Fig. 9a, the diffusion flame was positioned mostly at the side of the piston and between the intake and exhaust valve where the spray collided with the piston as illustrated in Fig. 7a for both G100 and G95L5. For G100, diffusion flame decreased as injection pressure increased since as injection pressure increase, wall film thickness decreases^29^. For G95L5, substantial diffusion flame was continuously observed regardless of injection pressure. To understand this phenomenon, other studies were examined based on the properties of the lubricant. From Bai et al.^30^, spray-wall interaction regimes are classified into stick, spread, rebound, and splash. Then, the transition criteria between theses regimes were calculated using droplet Laplace number and were compared with droplet Weber number to identify the possibilities of transition. Based on this article, we calculated that the lubricant droplet Laplace number is substantially lower than gasoline due to the high viscosity and high surface tension, which is proportional to viscosity^31^, while the droplet Weber numbers were similar. This means that the lubricant droplet tends to ‘stick’ or ‘spread’ significantly more than gasoline since the transition criteria are much higher than the droplet Weber number, resulting in an increase in wall film as the lubricant blend ratio increased. In addition, Yu et al.^32^ investigated the spray impingement to improve emissions during early injection timing using dimethyl ether (DME) blended diesel. The results showed that wall film average thickness and variation was due to impingement momentum, wall film mass and fuel properties. From this article, lubricant properties, such as kinematic viscosity, surface tension, and vapor pressure caused an increase in average wall film thickness. Therefore, after the spray collided with the piston surface, a wall film was formed on the piston surface without a thickness reduction, and it did not participate in the combustion process, causing diffusion flame as the combustion flame front contacted the wall film. At an injection timing of BTDC 270°, the diffusion flame at G100 decreased significantly compared to the diffusion flame at an injection timing of BTDC 330° as illustrated in Fig. 9b. As injection pressure increased, the diffusion flame notably decreased and could not be found at an injection pressure of 35 MPa. This was not the case for G95L5. The amount of diffusion flame did not decrease despite the increase in injection pressure. Also, diffusion flame was observed at the side of the piston, but it was also observed in the middle of the piston. These characteristics were also due to the properties of the lubricant and its influence of wall film development with the effect of in-cylinder flow and fuel behavior. Spray from the injector at an injection timing of BTDC 270 did not directly collide with the piston nor the cylinder liner, as described in Fig. 7b. Therefore, the wall film caused by spray impingement on the piston surface did not occur for this injection timing. However, from Lee et al.^33^, unevaporated fuel droplets contact the piston head and cylinder liner at this injection timing. Furthermore, high viscosity of the lubricant blended fuel caused fuel droplets to be stuck on the piston and cylinder surface. Also, low volatility prevented fuel droplets from evaporating resulting in the development of wall film. Then, this unburned wall film met the combustion flame front resulting in a diffusion flame as mentioned before. The effect of lubricant at an injection timing of BTDC 180° was also identified. G100 showed marginal amount of diffusion flame, and diffusion flame decreased as injection pressure increased. Diffusion flame was no longer visible at an injection pressure of 35 MPa resulting in a black image due to lack of chemiluminescent source. Whereas diffusion flame was still identified on G95L5 as shown in Fig. 9c. The cause of the diffusion flame for G95L5 was similar to the cause of diffusion flame at an injection timing of BTDC 270° but on a different position of the cylinder. At an injection timing of BTDC 180°, the spray direction was more towards the cylinder liner as illustrated in Fig. 7c. This was due to the upward in-cylinder flow at this injection timing^34^, which led unevaporated fuel droplets contact and stick on the cylinder liner creating wall film. As a result, the wall film at the cylinder liner was met with the combustion flame front, causing diffusion flame. From these results, difference in diffusion flame amount implied that lubricant blended in the gasoline affected wall film amount which is one of the causes of particle formation.

### Effect of gasoline-lubricant blend ratio on particulate emission

**Figure 10:**
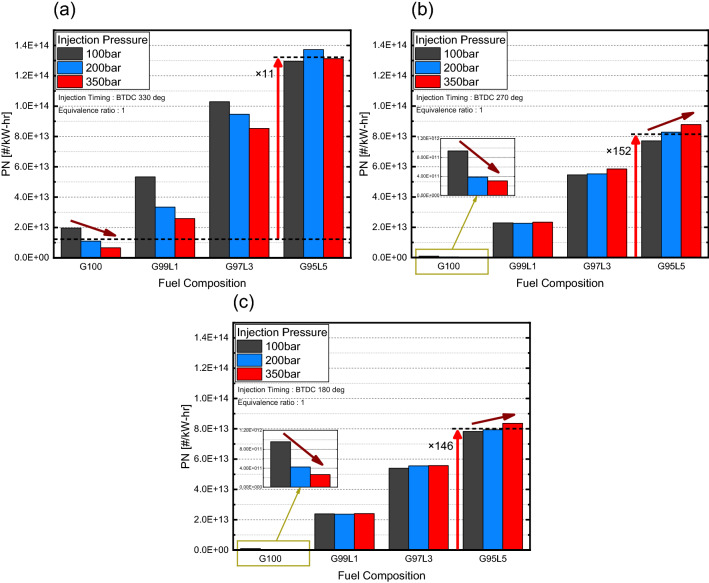
PN emissions with respect to injection pressure and lubricant blend ratio.

PN emissions emitted as a function of lubricant blend ratio are shown in Fig. 10. For G100, the overall PN decreased significantly as injection pressure increased for all injection timings due to a reduction in the wall film. However, as the weight percent of lubricant increased, the results deviated from G100. At an injection timing of BTDC 330° at G95L5, the overall PN was 11 times larger than G100. Also, the G95L5 lubricant blend ratio resulted in no reduction in PN with respect to injection pressure as shown in Fig. 10a. From the investigation above, the overall increase in PN was affected by an increase in unburned fuel remaining on the piston surface and cylinder liner, creating a wall film and a causing pool fire, which is a dominant feature of PN emissions from GDI engines. These phenomena were due to the lubricant properties. The high viscosity and surface tension of the lubricant resulted in a high transition criterion from stick to splash, which makes regime transition difficult even with increased droplet velocity at higher injection pressure. This did not reduce the wall film, resulting in similar PN regardless of injection pressure. For an injection timing of BTDC 270°, the overall increase in PN emissions at G95L5 was 152 times that of G100. While G100 PN emissions decreased as injection pressure increased from better atomization and evaporation of fuel droplets, PN emissions increased with respect to injection pressure, and the rate of increase became more significant as the lubricant blend ratio increased as shown in Fig. 10b. The substantial overall increase in PN emissions was also due to the properties of the lubricant. Since the spray in this injection timing did not collide with the piston nor the cylinder liner, overall PN emissions were low for G100. However, for the lubricant blended fuel, fuel droplets that did not evaporate due to the low volatility stuck to the piston surface and cylinder liner, resulting in the observed difference in PN emissions. The increase in PN emissions on injection pressure and lubricant blend ratio was from the increase in spray momentum, which is proportional to injection pressure. As spray momentum increased from boosted injection pressure, the spray penetration length also increased, which led to an increase in unevaporated fuel droplets reaching the piston surface and cylinder liner. This phenomenon becomes more significant as the lubricant blend ratio increases, resulting in an increase in PN with respect to lubricant blend ratio. Overall PN emissions at an injection timing of BTDC 180° increased significantly, i.e., 146 times moving from G100 to G95L5. Furthermore, the rate of PN generation, increased with respect to injection pressure and lubricant blend ratio as shown in Fig. 10c. The mechanism that influenced the overall increase in PN was the same as the mechanism at an injection timing of 270°. However, the rate of increase was lower than at an injection timing of BTDC 270° since the spray momentum was the only main source for the unevaporated lubricant droplet to move to the cylinder liner.

**Figure 11:**
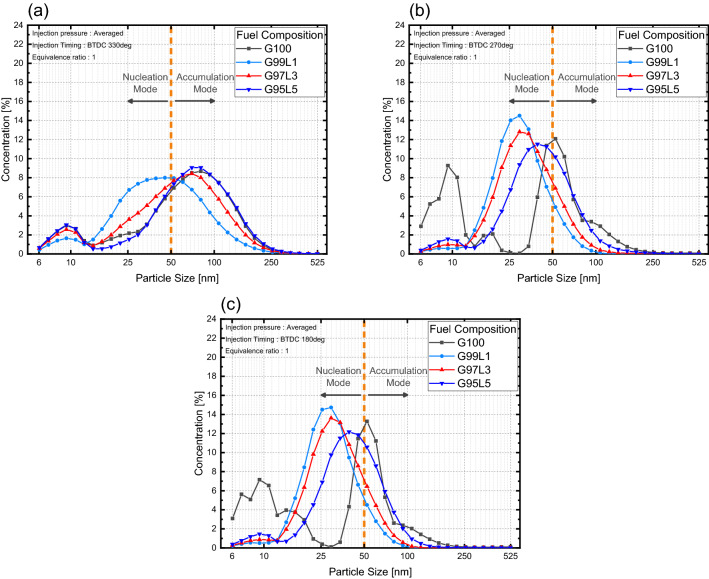
Particle size distributions with respect to lubricant blend ratio.

**Figure 12:**
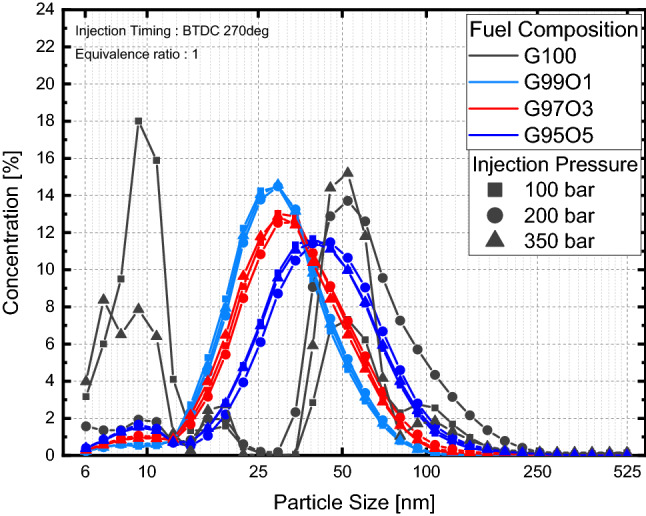
Particle size distributions with respect to injection pressure and lubricant blend ratio.

Particle size distributions were also influenced by lubricant blend ratio as shown in Fig. 11. Here, particle size distributions with respect to lubrication blend ratio for various injection pressures were similar as shown in Fig. 12. Therefore, particle size distributions on injection pressure were averaged to clearly see the influence of lubricant blend ratio at different injection timings. For an injection timing of BTDC 330**°**, nucleated particles increased compared to G100 at G99L1, but the overall particle size shifted towards accumulation mode as the lubricant blend ratio increased as illustrated in Fig. 11a. Size distributions were similar for all lubricant blend ratios. The increase in nucleation particles when lubricant was blended was due to the influence of lubricant properties as investigated by Amirante et al.^20^ The increase in overall size in relation to the lubricant blend ratio (except G100) was due to an increase in nucleation particles from an increase in wall film. Nucleation particles collided with each other, forming larger particles by agglomeration^35^. The increase in nucleation particles resulted in a higher probability of nucleated particles colliding with each other, which increased particle size. The change in overall particle size for injection timings of BTDC 270 and 180 were similar to the overall change in an injection timing of BTDC 330**°**, but the shift towards accumulation mode was lower as presented in Fig. 11b,c. This was due to the reduction in the wall film compared to BTDC 330**°**, which reduced the number of nucleated particles. Size distributions at both injection timings were distinct from G100. G100 size distributions were divided into nucleation mode and accumulation mode, whereas lubricant blended fuels size distributions were clustered near the boundary between nucleation mode and accumulation mode. Particle formation in G100 was mainly due to the mixture homogeneity, whereas particle formation for the lubricant blended fuel was primarily due to the wall film, which can be further explained by the size distributions at BTDC 330**°**.

## Conclusion

Experiments were conducted to identify how particulate matter formation was influenced by lubricants and how these affected PN and particle size distributions using a single-cylinder GDI combustion and optical engine fueled by different lubricant gasoline blend ratios. Our results are summarized as follows.

1. Lubricant did not affect mixture homogeneity for all cases. Combustion characteristics on various lubricant blend ratio were similar, which is one of the major parameters distinguishing mixture homogeneity. Also, CO emissions showed no trends, indicating that lubricant blend ratio did not affect CO emissions. NOx emissions seemed to be affected by the lubricant, but this was a measurement error based on combustion characteristics and had no relationship with CO emissions. Since CO and NOx relationships also have a major influence on mixture homogeneity, this implies that there was no influence of lubricant on the mixture homogeneity. Furthermore, spray images showed similar results between G100 and G95L5 in terms of spray behavior, which also indicated there was no difference in mixture homogeneity from the lubricant.
2. The wall film was a major contributor to particulate emissions for various lubricant blend ratios. An increase in fuel consumption and THC emissions indicated that the lubricant had an influence on unburned fuels. Furthermore, diffusion flame was constantly observed for G95L5 regardless of injection conditions, whereas diffusion flame decreased for G100 as injection pressure increased and injection timing retarded. These different results for G100 and G95L5 showed that the lubricant had an influence on wall films. This was due to the extremely low volatility and high viscosity of the lubricant compared to gasoline, which increased unevaporated fuel droplets. When these droplets were in contact with the piston or in-cylinder liner, it developed into a wall film, which ignited to form diffusion flame due to incomplete combustion, resulting in an increase in unburned fuels and the formation of particulate matter.
3. PN emissions and particle size distribution characteristics with respect to lubricant blend ratio demonstrated the influence of the lubricant on the mechanism of wall film formation. For an injection timing of BTDC 330°, where the spray impinges on the piston surface, lubricant properties increased the wall film, which boosted PN emissions and nucleated particles. Also, it affected the amount of wall film, resulting in similar PN emissions with respect to injection pressure. For an injection timing of BTDC 270 and 180°, where spray does not directly collide with either the piston or in-cylinder liner, lubricant properties increased unevaporated fuel droplets, which created a wall film inside the combustion chamber. This resulted in an increase in PN emissions and differences in particle size distributions. In addition, increasing the injection pressure helped fuel droplets reach the piston and/or in-cylinder liner, resulting in a proportional relationship between PN emissions and injection pressure.

## Data Availability

The data that support the experimental results in this study are available from the corresponding author upon reasonable request.

## Abbreviations

PN: Particle number
ATDC: After top dead center
BTDC: Before top dead enter
ROHR: Rate of heat release
CA: Crank angle
CO: Carbon monoxide
NOx: Nitrous oxides
THC: Total hydrocarbon
ASOI: After start of injection
ASOS: After start of spark ignition
